# Decoding tumor heterogeneity in uveal melanoma: basement membrane genes as novel biomarkers and therapeutic targets revealed by multi-omics approaches for cancer immunotherapy

**DOI:** 10.3389/fphar.2023.1264345

**Published:** 2023-09-26

**Authors:** Yunyue Li, Huabao Cai, Jinyan Yang, Xixi Xie, Shengbin Pei, Yifan Wu, Jinhao Zhang, Guobin Song, Jieying Zhang, Qinhong Zhang, Hao Chi, Guanhu Yang

**Affiliations:** ^1^ Queen Mary College, Medical School of Nanchang University, Nanchang, China; ^2^ Department of Neurosurgery, First Affiliated Hospital of Anhui Medical University, Hefei, China; ^3^ School of Stomatology, Southwest Medical University, Luzhou, China; ^4^ Department of Breast Surgery, The First Affiliated Hospital of Nanjing Medical University, Nanjing, China; ^5^ First Teaching Hospital of Tianjin University of Traditional Chinese Medicine, Tianjin, China; ^6^ Heilongjiang University of Chinese Medicine, Harbin, China; ^7^ Clinical Medical College, Southwest Medical University, Luzhou, China; ^8^ Department of Specialty Medicine, Ohio University, Athens, OH, United States

**Keywords:** uveal melanoma, basement membrane genes, machine learning, multi-omics, tumor heterogeneity, cancer immunotherapy

## Abstract

**Background:** Uveal melanoma (UVM) is a primary intraocular malignancy that poses a significant threat to patients’ visual function and life. The basement membrane (BM) is critical for establishing and maintaining cell polarity, adult function, embryonic and organ morphogenesis, and many other biological processes. Some basement membrane protein genes have been proven to be prognostic biomarkers for various cancers. This research aimed to develop a novel risk assessment system based on BMRGs that would serve as a theoretical foundation for tailored and accurate treatment.

**Methods:** We used gene expression profiles and clinical data from the TCGA-UVM cohort of 80 UVM patients as a training set. 56 UVM patients from the combined cohort of GSE84976 and GSE22138 were employed as an external validation dataset. Prognostic characteristics of basement membrane protein-related genes (BMRGs) were characterized by Lasso, stepwise multifactorial Cox. Multivariate analysis revealed BMRGs to be independent predictors of UVM. The TISCH database probes the crosstalk of BMEGs in the tumor microenvironment at the single-cell level. Finally, we investigated the function of ITGA5 in UVM using multiple experimental techniques, including CCK8, transwell, wound healing assay, and colony formation assay.

**Results:** There are three genes in the prognostic risk model (ADAMTS10, ADAMTS14, and ITGA5). After validation, we determined that the model is quite reliable and accurately forecasts the prognosis of UVM patients. Immunotherapy is more likely to be beneficial for UVM patients in the high-risk group, whereas the survival advantage may be greater for UVM patients in the low-risk group. Knockdown of ITGA5 expression was shown to inhibit the proliferation, migration, and invasive ability of UVM cells in vitro experiments.

**Conclusion:** The 3-BMRGs feature model we constructed has excellent predictive performance which plays a key role in the prognosis, informing the individualized treatment of UVM patients. It also provides a new perspective for assessing pre-immune efficacy.

## 1 Introduction

Uveal melanoma (UVM) is a rare yet aggressive primary intraocular malignancy arising from ocular melanocytes, constituting a small fraction of all melanomas (Singh et al., 2005; Singh et al., 2011; Chattopadhyay et al., 2016; Chi et al., 2022). It imposes significant threats to patients’ visual function and overall survival, with a high mortality rate of up to 50% attributed to its severe clinical presentation, malignancy, and limited treatment options (Andreoli et al., 2015). Notably, UVM exhibits a propensity for metastasis, with approximately half of the patients developing distant organ metastases, most commonly involving the liver, lung, and bone (Rusňák et al., 2020). Unfortunately, current therapeutic modalities for UVM have shown limited efficacy in managing metastatic disease (Augsburger et al., 2009; Damato, 2018). As a result, recent research endeavors have focused on the development of targeted therapeutics and immunotherapeutic strategies, including immune checkpoint inhibitors, vaccines, and adoptive cell therapy, to address the unmet medical needs in UVM (Curran et al., 2010; Larkin et al., 2015; Bol et al., 2016). However, the underlying etiology and molecular mechanisms driving UVM remain largely elusive (Smit et al., 2020; Derrien et al., 2021; Katopodis et al., 2021). Consequently, there is a critical need to identify novel prognostic biomarkers and molecular targets that can accurately predict patient outcomes and facilitate personalized treatment approaches, ultimately improving the quality of life for individuals affected by UVM.

The basement membrane (BM) is a specialized extracellular matrix located at the basal aspect of epithelial tissues, primarily composed of collagen IV, laminin, heparan sulfate proteoglycans, BM-40, and nidogen (Timpl, 1989). Its crucial role in establishing and maintaining cellular polarity and providing mechanical support to tissues is well-recognized (Banerjee et al., 2022). Moreover, BMs play critical roles in various physiological processes, including embryonic development, organ morphogenesis, and adult tissue homeostasis (Li et al., 2003). Perturbations in BM protein expression and turnover have been implicated in tumorigenesis, and dysregulation of BM integrity has been associated with tumor metastasis (Valastyan and Weinberg, 2011; Naba et al., 2014). While BM-related genes have shown prognostic significance in several cancers, their role, and prognostic implications in uveal melanoma (UVM) remain poorly understood. To elucidate the immunological status of UVM patients and accurately predict prognosis, this study aimed to develop a novel risk-scoring system based on BM-related genes. The objective was to establish a theoretical foundation for personalized therapeutic interventions tailored to individual patients. By comprehensively characterizing the expression and functional relevance of BMRGs, this risk-scoring system would enable precise prognostic stratification and facilitate tailored treatment strategies in UVM.

Following the rapid advancement of bioinformatics (Song et al., 2022a; Zhao et al., 2022a; Jin et al., 2022), a considerable amount of research has been conducted to establish models for predicting the prognosis of UVM through machine learning. For example, Zheng et al. established an autophagy-related gene (ARG) risk model and validated it with TCGA and four external independent UVM cohorts, revealing that UVM patients with higher risk scores exhibited higher levels of immune cell infiltration and enrichment of tumor markers (Zheng et al., 2021); Lv et al. constructed a UVM prognostic model based on the Epithelial-mesenchymal transition (EMT) signature, which found that patients with high EMT scores potentially had higher response rates to immunotherapy (Lv et al., 2022); Yang et al. utilized immune markers systematically to develop a prognostic six-immune-gene signature via RNA sequencing data from TCGA and GEO databases for predicting the overall survival outcome of UVM patients (Yang et al., 2023). Meanwhile, several studies have reported that BMRG signatures could predict the prognosis of tumor survivors and provide a potential target for immunotherapy (Cai et al., 2022; Shen et al., 2023). However, BMRG-related models have not yet been established and validated for prognostic prediction in UVM patients (Song et al., 2022a).

In this study, we developed a prognostic model for UVM using the TCGA-UVM cohort. We carefully selected three reliable basement membrane-related genes (BMRGs) through a rigorous screening process and employed two machine learning techniques to construct the model. By integrating genetic information from UVM patients, we aimed to explore the prognostic value of these three BMRGs and develop novel tools to enhance therapeutic strategies. Our analysis involved assessing the interaction between BMRGs and the immune microenvironment, as well as evaluating the impact of BMRGs on immunotherapy and chemotherapy sensitivity. We eventually verified the functional role of the ITGA5, the gene with the highest absolute HR value, in UVM cells by an *in vitro* experiment. By leveraging advanced computational methods and integrating multi-dimensional data, we sought to gain insights into the role of BMRGs in determining the prognosis of UVM, identify potential avenues for improving treatment regimens, and offer possibilities for developing personalized therapeutic approaches. These findings have the potential to enhance patient outcomes and pave the way for further advancements in UVM research and clinical practice.

## 2 Materials and methods

### 2.1 Patient data sources

We utilized the TCGA-UVM cohort, obtained from the publicly available TCGA database, as our training set, consisting of gene expression profiles and clinical data from 80 tumor patients. To ensure accurate analysis, we performed preprocessing steps on the data. Initially, we converted the level 3 HTSeq-fragments per kilobase (FPKM) data into transcripts per million reads (TPM) to account for gene length and sequencing depth variations across samples. This conversion was done using a formula that normalized the TPM values. Subsequently, we applied a logarithmic transformation to the TPM values to normalize the data and enhance comparability between samples. It is important to note that due to significant variation in sample sizes among UVM patients at stages M and N, these stages were excluded from our analysis to ensure robustness and reliability. Furthermore, we incorporated two external validation datasets, GSE84976 and GSE22138, from the GEO database. It is worth noting that datasets GSE84976 and GSE22138 were merged together to act as a validation set, and in order to mitigate the effects of batch differences between the microarray expression data, we utilized the ComBat function in the R package “sva” to achieve correction for batch effects. For comprehensive details on these datasets, see Supplementary Material. These datasets included genetic profiles and clinical data from 56 UVM patients, and their inclusion aimed to enhance the validity and generalizability of our analyses. In the training cohort, we transcribed and analyzed tissue samples from eye cancer patients for comparative analyses to obtain genes that were aberrantly expressed in eye cancer patients. While in the external validation set, we only included samples from eye cancer patients analyzed. In addition, we required complete patient follow-up and clinical information in the cohort and complete micro-matrix data in the cohort to ensure data quality for subsequent bioinformatics analysis. By employing these rigorous preprocessing steps and integrating multiple datasets, we aimed to improve the accuracy and reliability of our findings, providing valuable insights into the molecular characteristics and clinical implications of UVM.

### 2.2 Consensus clustering analysis

To gain deeper insights into the mechanistic implications of BMRGs in UVM, we employed advanced analytical methodologies. The “Consensus Cluster Plus” R package (Zhao et al., 2022b; Wang et al., 2022) was leveraged to classify UVM patient samples into distinct clusters based on the expression patterns of BMRGs, thereby unveiling unique gene expression profiles associated with specific subtypes. Differential expression patterns of BMRGs across clusters, along with clinicopathological parameters, were visualized using the “pheatmap” R package (Bhattacherjee et al., 2019; Song et al., 2022b). To elucidate the distinct biological pathways and processes underlying these clusters, we retrieved the “c2. cp.kegg.v7.4. symbols.gmt” file from the MSigDB database (Liberzon et al., 2015) for genomic variation analysis via GSVA. Employing the “GSVA” R package (Hänzelmann et al., 2013), we systematically analyzed pathway differences between clusters, revealing noteworthy disparities in key pathways among diverse UVM subtypes. Furthermore, the Single Sample Genome Enrichment Analysis (ssGSEA) algorithm (Zhuang et al., 2021; Huang et al., 2023) was applied to assess the infiltration levels of immune cells and expression levels of immune checkpoints within the identified clusters. This integrative approach shed light on potential variations in the immune microenvironment across UVM subtypes, offering crucial insights into the prospective efficacy of immune checkpoint-based therapies in specific patient cohorts.

### 2.3 Model construction and validation

The dataset of basement membrane (BM) genes was obtained from the Basement Membrane BASE database (https://bmbase.manchester.ac.uk), comprising a comprehensive collection of 224 genes associated with the basement membrane protein. To explore the potential prognostic relevance of these genes, univariate Cox regression analysis was performed, resulting in the identification of 81 genes significantly associated with survival outcomes. To further refine the gene set and mitigate the risk of overfitting, we employed the LASSO (Least Absolute Shrinkage and Selection Operator) method, a powerful machine learning approach (Chi et al., 2023a; Chi et al., 2023b). The “glmnet” R package (Engebretsen and Bohlin, 2019; Ren et al., 2023) was utilized to implement LASSO, which involves adding a penalty term to the regression model. This penalty encourages the coefficients of less influential predictors to shrink toward zero, effectively selecting the most informative subset of predictors. By applying LASSO, we successfully narrowed down the candidate genes to eight. Subsequently, a stepwise multi-factor Cox regression model was employed to identify and estimate the coefficients of the core genes from the selected set. Through this iterative procedure, we ultimately derived a risk profile consisting of four BMRGs. For each patient, the risk score was calculated by combining the expression levels of these genes with their corresponding coefficients: Risk score = ExpressionmRNA1 × CoefmRNA1 + ExpressionmRNA2 × CoefmRNA2 + ExpressionmRNAn × CoefmRNAn. By leveraging these analytical approaches, we aimed to establish a robust and concise set of BMRGs with prognostic implications in order to facilitate risk stratification and inform personalized treatment strategies for patients.

### 2.4 Correlation between clinicopathological features and risk scores

Investigating the relationship between risk scores and relevant clinical features in patients with uveal melanoma (UVM) can provide valuable insights for clinical prognostic assessment. To visualize the associations between clinical features and the modeled genes, we employed the “pheatmap” R package (Lu et al., 2021) to generate heat maps displaying multiple groups of clinical features. To gain a deeper understanding of the differences in risk scores among various patient subgroups, we performed clinical analyses on the entire sample cohort. The patients were stratified based on different clinical characteristics, including age (≤65 and >65 years), sex (male and female), pathological stage (II and III-IV), and T-stage (T2 and T3-4). Between-group differences were assessed using the “ggpubr” package (Whitehead et al., 2019). By examining the relationships between risk scores and clinical subgroups, we aimed to identify potential variations in risk profiles based on different demographic and pathological factors. These analyses would contribute to a more comprehensive understanding of the prognostic implications of risk scores in UVM patients and their clinical relevance.

### 2.5 Independent prognostic analysis and nomogram construction

To evaluate the independent prognostic value of the risk score in predicting uveal melanoma (UVM) outcomes, we conducted both univariate and multivariate Cox regression analyses. These analyses aimed to assess whether the risk score could serve as a reliable prognostic factor, independent of conventional clinicopathological characteristics. The “rms” R package (Zhang et al., 2022) was employed to construct a nomogram incorporating the risk score and clinicopathological features. This nomogram provided a visual tool for predicting the survival of patients in the TCGA-UVM cohort, enabling clinicians to estimate individual patient prognoses more accurately. To assess the predictive performance of the nomogram, we utilized the “ggDCA” R package (Mao et al., 2021) to develop decision curve analysis (DCA) and calibration curves. The DCA allowed us to evaluate the clinical benefits of using the nomogram compared to other predictive models or strategies. Calibration curves were generated to assess the calibration accuracy of the nomogram in predicting patient survival. These comprehensive analyses aimed to validate the prognostic value of the risk score and provide clinicians with a practical tool for prognostic assessment in UVM patients. By integrating the risk score with clinicopathological characteristics, the nomogram offered improved prognostic accuracy, ultimately enhancing patient management and treatment decision-making.

### 2.6 Establishing the equations for signatures

After scoring all UVM patients based on the risk model equation, we determined the median risk score using the ‘survminer’ R package. Subsequently, we categorized the patients into a low-risk group and a high-risk group. Survival curves were plotted for both groups to visually compare their survival outcomes. To evaluate the predictive performance of the risk model, we calculated the C-index using the ‘pec’ R package. The C-index provides a measure of concordance between predicted risk scores and actual survival outcomes. To further assess the predictive power of the genetic traits, we conducted an analysis of receiver operating characteristic (ROC) curves using the ‘time-ROC’ R package. ROC curves allow us to evaluate the sensitivity and specificity of the genetic traits in predicting survival outcomes. Additionally, decision curve analysis (DCA) was performed for the multi-factor Cox regression model using the ‘ggDCA’ R package. DCA provides insights into the clinical utility of the predictive model by assessing the net benefits of different strategies or models across a range of threshold probabilities. Through these analyses, we aimed to assess the predictive accuracy and clinical usefulness of the risk model in UVM patients. The survival curves, C-index, ROC curves, and DCA plots provide valuable information for understanding the prognostic value and potential application of genetic traits in UVM patient management and treatment decision-making.

### 2.7 Enrichment analysis

To analyze the Gene Ontology (GO) pathway, we utilized the “ClusterProfiler” R package (Song et al., 2023; Zhang et al., 2023). In the generated graphs, a *p*-value of less than 0.05 indicated a statistically significant difference, highlighting the enriched pathways and functional categories associated with the genes of interest. For further enrichment analysis, we conducted GSVA using the “GSVA” R package. The data from “c2. cp.kegg.v7.5.1. symbols.gmt” in the MSigDB database were utilized to explore the functional annotation and enrichment pathways. To visualize the results, heatmaps were generated using the ‘heatmap’ R package. Adjusted *p*-values of less than 0.05, obtained through the ‘limma’ R package, indicated the statistical significance of subgroup differences in the heatmap. Through functional enrichment analysis, we aimed to gain insights into the biological functions, pathways, and processes associated with differentially expressed genes related to BMRGs in UVM. These analyses contribute to a better understanding of the molecular mechanisms underlying UVM and provide valuable information on functional annotations and enriched pathways associated with BMRGs in the context of UVM.

### 2.8 Immuno-infiltration analysis

Multiple methods have been developed to quantify immune infiltration scores, including XCELL, TIMER, QUANTISEQ, MCPCOUNT, EPIC, CIBERSORT, and CIBERSORT-ABS. These methods offer diverse approaches for evaluating the presence and abundance of immune cells within the tumor microenvironment. To investigate the association between immune cells and risk scores, Spearman correlation analysis was employed, allowing for a comprehensive understanding of the immune landscape in UVM. Utilizing the immune cell profiles of UVM patients, we applied the ssGSEA method to stratify patients into distinct low- and high-risk groups based on their immune signatures. Furthermore, we examined the differential expression of 20 suppressive immune checkpoints between the identified high-risk and low-risk groups, shedding light on the potential influence of immune checkpoint blockade therapies. To assess and visualize the impact of immunotherapy in UVM patients, we utilized the widely adopted ‘limma’ and ‘ggpubr’ R packages. To expand our understanding of the genetic underpinnings related to cancer and immunity, we referred to the curated collection of genes provided by Xu et al., available on their website (Xu et al., 2018). Employing the R package “ggcor,” we explored the correlation between risk scores and these two genetic traits, unraveling potential associations between genetic alterations and disease prognosis in UVM. Additionally, to predict immune infiltration estimates and immunotherapy response data, we leveraged the computational tool ImmuCellAI (Miao et al., 2020). This powerful resource enables comprehensive analyses of the immune landscape and aids in guiding immunotherapeutic strategies for UVM patients.

### 2.9 TISCH analysis

The Tumor Immunological Single Cell Centre (TISCH) hosts a comprehensive single-cell RNA sequencing database that focuses on investigating the intricate tumor microenvironment (TME). This valuable resource facilitates detailed annotation of various single-cell types, enabling in-depth analysis of gene expression within distinct cellular populations. By examining gene expression patterns across different cell types, we can unravel the intricate variations within the tumor microenvironment of individual UVM patients, thus shedding light on the underlying heterogeneity of UVM. This comprehensive characterization of the TME aids in elucidating the complex dynamics and functional implications of different cell types within the UVM context.

### 2.10 Cell culture

The human uveal melanoma cells (MuM-2B, OCM-1) utilized in this investigation were generously provided by the Cell Resource Center at Shanghai Life Sciences Institute. These cells were cultivated under controlled conditions in Dulbecco’s Modified Eagle’s Medium (DMEM) (Gibco, United States), supplemented with 1% penicillin/streptomycin and 10% fetal bovine serum (FBS) (Gibco, United States), within a humidified incubator set at 37°C with a 5% CO2 atmosphere.

### 2.11 CCK-8 assay

To assess the impact of ITGA5 on the proliferative capacity of uveal melanoma (UVM) cells, the Cell Counting Kit-8 (CCK-8) assay was employed. UVM cells were cultured in 96-well microplates in triplicate, with each well initially seeded with 5,000 cells. Subsequent to transfection, the cells were subjected to treatment at 37°C for a duration of 2 h, utilizing 10 μL of CCK-8 solution (A311-01, vazyme, Nanjing, China) mixed with 90 μL of complete media in each well at specific time points (0, 24, 48, 72, or 96 h). Following the respective incubation periods, the absorbance of each well was quantified at 450 nm using a microplate reader.

### 2.12 Wound-healing assay

The wound healing assay was employed to evaluate the migratory behavior of MuM-2B and OCM-1 cells, providing valuable insights into their migratory patterns. The transfected cells were cultured in a six-well plate and incubated at 37°C until they reached approximately 80% confluence. To create a standardized wound, a sterile 200 μL pipette tip was carefully used to generate a linear scrape across the cell monolayers. Following this, the medium was replaced with serum-free medium after two washes with phosphate-buffered saline (PBS) to eliminate any cellular debris. The movement of cells into the wound area was monitored at 0 h and 48 h using an inverted microscope (Olympus, Japan), enabling the quantification of the distance traveled by the cells into the wound surface.

### 2.13 Transwell assay

Cell migration was assessed using the Transwell migration assay, which involved a 24-well Transwell plate equipped with 8 μm-pore membrane filters. Briefly, the bottom chamber of the Transwell plate was supplemented with media containing 10% fetal bovine serum (FBS), while the top chamber was coated with 2 × 10^5 cells suspended in serum-free medium. Following a 48-h incubation period, the cells that had migrated to the bottom chamber were fixed in 4% methanol for 10 min and subsequently stained with 0.1% crystal violet (Solarbio, Beijing, China) for 15 min.

### 2.14 Statistical analysis

The statistical analysis was conducted using R software version 4.1.3. To compare the overall survival (OS) between the high-risk and low-risk groups, Kaplan-Meier (KM) survival curves and log-rank tests were employed. In addition, Lasso regression analyses were performed to assess the potential relevance of BMRGs. A stepwise multivariate Cox regression analysis was then employed to construct a BMRG signature. The predictive performance of the model was evaluated using a time-dependent ROC curve. The relationship between the risk score and immune cell infiltration was assessed using Spearman correlation analysis. To compare the ratios of tumor immune infiltrating cells (TIIC), immunological checkpoints, and immune function between the two groups, the Wilcox test was applied. Statistical significance was determined by *p*-values <0.05, and a false discovery rate (FDR) < 0.05 was considered statistically significant. The CCK-8 data analysis was conducted using GraphPad Prism Software version 8.3.0. The mean values ±standard deviation (SD) were determined based on data obtained from three independent experiments. Statistical significance was assessed using analysis of variance (ANOVA), with a significance level set at *p* < 0.05.

## 3 Results

### 3.1 Consensus clustering determined the molecular subtypes of BMRGs

**FIGURE 1:**
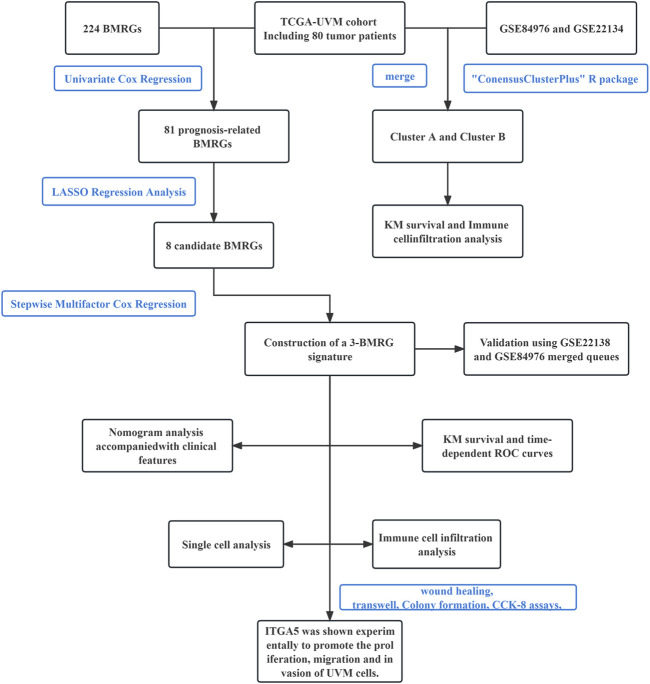
Flow chart of this study.

**FIGURE 2:**
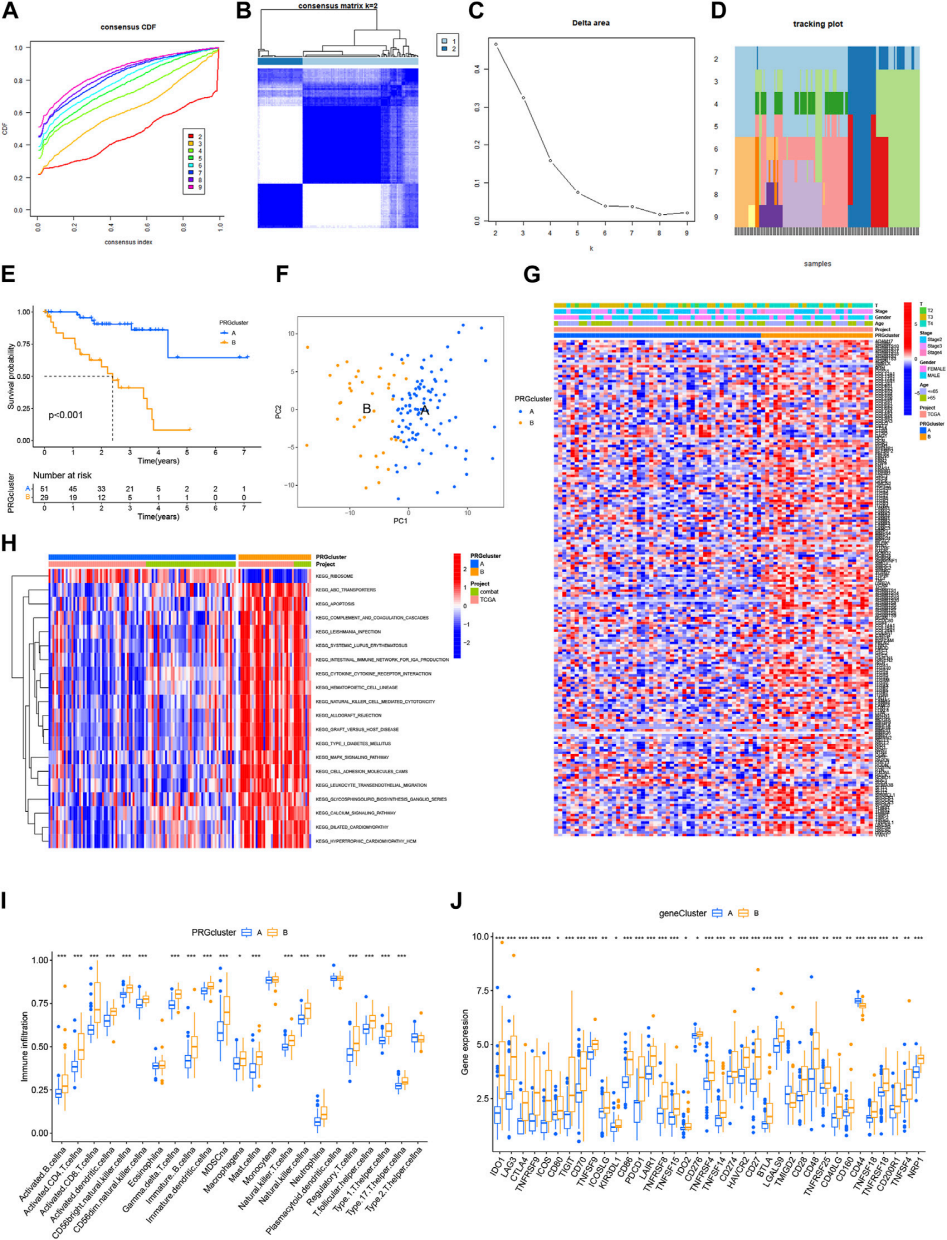
Consensus clustering determined the molecular subtypes of BMRGs. **(A)** Consensus clustering CDF with K = 2 to 9. **(B)** Consensus matrix heatmap for K = 2 clusters. **(C)** CDF plot illustrating the consensus clustering results for K = 2 to 9. **(D)** Tracking plot displaying the sample classification across K = 2 to 9 clusters. **(E)** Kaplan-Meier survival curves comparing the survival outcomes between Cluster A and Cluster B. **(F)** Principal component analysis (PCA) plot visualizing the distribution of samples. **(G)** Correlation analysis depicting the relationship between BMRGs expression and clinicopathological parameters. **(H)** Enrichment analysis of KEGG pathways in Cluster A and Cluster B. **(I)** Comparison of immune cell infiltration levels between clusters. **(J)** Differential expression of immune checkpoints between Cluster A and Cluster B. Statistical significance: *p < 0.05, **p < 0.01, ***p < 0.001, ns > 0.05.

The primary study design is presented in Figure 1, illustrating the overall flow of the investigation. The cumulative distribution function (CDF) values demonstrated an increasing trend in relation to the consensus index, indicating successful classification. To assess cluster composition and quantity, the consensus matrix serves as an excellent visual tool. We generated a color-coded heat map based on the consensus matrix, which revealed higher intra-cluster correlations and lower inter-cluster correlations when considering k = 2. These findings strongly support the acceptance of two subtypes (Cluster A and Cluster B) for categorizing UVM patients. Based on the CDF curves and the Delta area, k = 2 represents the optimal point to achieve maximal inter-cluster differences as the clustering index “k” increases from 2 to 9. Consequently, we divided the UVM patients into two subgroups (Figures 2A–D).

Furthermore, we investigated the differential survival prognosis across clusters using the Cluster Survival R package. The results indicated that patients in cluster A exhibited significantly better survival prognoses than those in cluster B (*p* < 0.001) (Figure 2E).

Principal component analysis (PCA) was performed to visualize risk distribution among different patient groups. The PCA plot (Figure 2F) demonstrated distinct differences between Cluster A and Cluster B patients. Additionally, we conducted further analysis to explore metabolic variations in BMRGs between clusters A and B. The heat map revealed notable expression differences and clinical traits associated with BMRGs in cluster B (Figure 2G).

To investigate potential biological pathways, we performed an enrichment analysis using the Kyoto Encyclopedia of Genes and Genomes (KEGG) pathway database on the clustered samples. We explored correlations among various cancer-related pathways, such as apoptosis, transporters, and the MAPK signaling pathway (Figure 2H). Moreover, we employed the ssGSEA algorithm to assess the distribution and correlation of 23 tumor-infiltrating immune cells (TIICs) to guide immunotherapy. Notably, cluster B exhibited higher levels of immune cell infiltration compared to cluster A (Figure 2I). Considering the critical role of immune checkpoints in tumor immunotherapy effectiveness and their prominence within the tumor microenvironment (TME), we evaluated immune checkpoint expression between the two patient clusters. The analysis revealed significantly upregulated immune checkpoint expression in Cluster B patients, except for TMIGD2 and CD44. Based on these findings, we conclude that Cluster B demonstrates a more favorable response and effectiveness toward immunotherapy (Figure 2J).

### 3.2 Development and validation of the BMRGs signature

**FIGURE 3:**
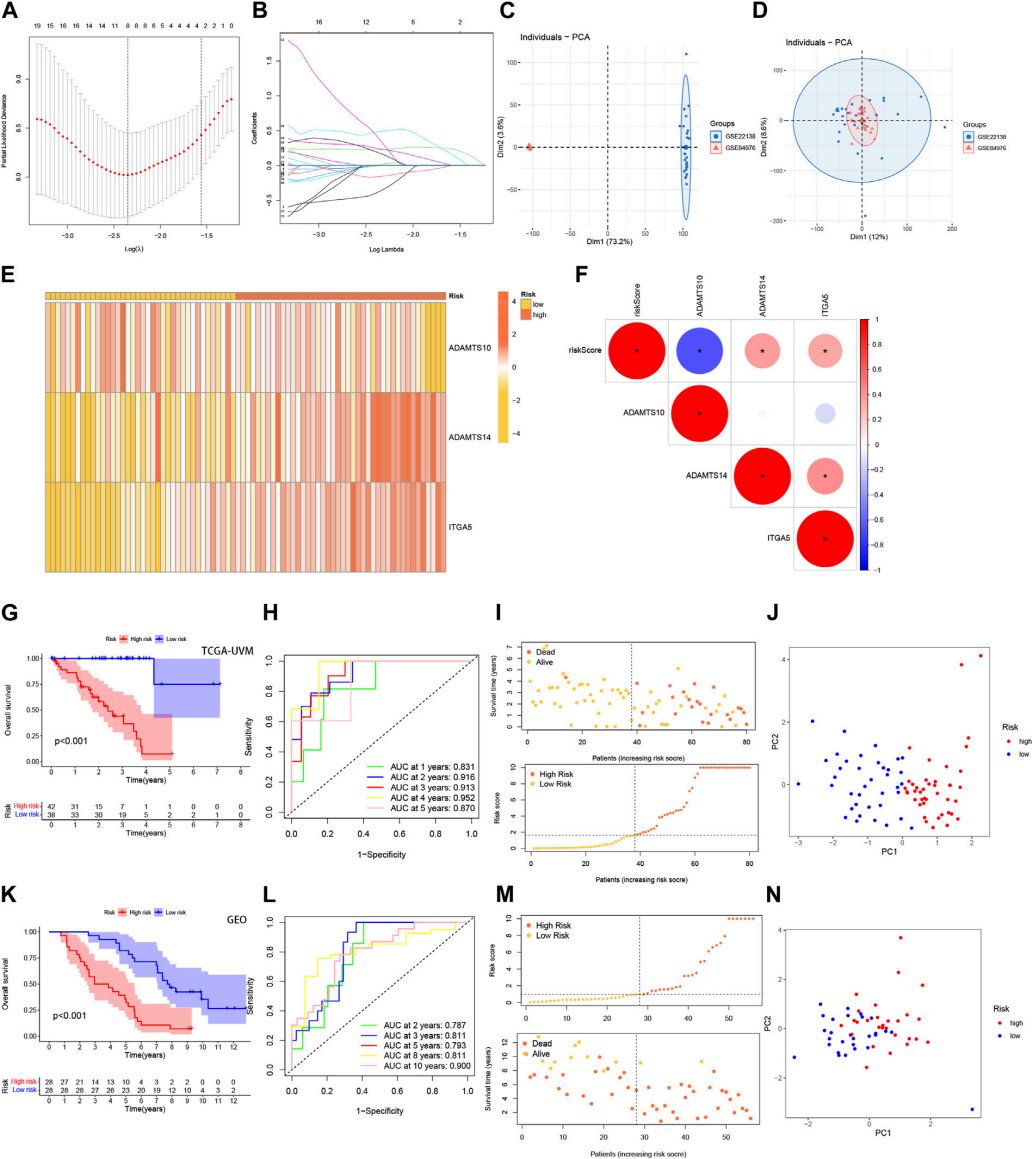
Development and validation of the BMRGs signature. **(A)** Ten-fold cross-validation for parameter selection using the LASSO model. **(B)** Profiles of LASSO coefficients. **(C)** Principal component analysis (PCA) plot of GSE22138. **(D)** PCA plot of GSE84976 after removing batch effects using Combat. **(E)** Heatmap illustrating the risk factors in high- and low-risk patients. **(F)** Correlation between three BMRGs and the risk score. **(G, K)** Kaplan-Meier curves comparing overall survival between low- and high-risk groups in the TCGA-UVM cohort and the GSE84976 cohort. **(H, L)** Time-dependent receiver operating characteristic (ROC) curves analysis of the TCGA-UVM cohort and the GSE84976 cohort. **(I, M)** Distribution of risk scores and survival status of UVM patients in the low- and high-risk groups in the TCGA-UVM cohort and the GSE84976 cohort. **(J, N)** PCA plot of the TCGA-UVM cohort and the GSE84976 cohort.

We developed a risk score model based on BMRGs to identify prognostic biomarkers in UVM patients. Differentially expressed BMRGs with prognostic value were selected using LASSO regression analysis, and the resulting LASSO regression curves and cross-validation plots are shown in Figures 3A,B, respectively. To address batch effects between GSE22138 and GSE84976 datasets, we employed the R package “Combat” for batch effect removal (Figures 3C,D). The prognostic index (PI) was calculated using the formula (−0.974 * ADAMTS10 exp.) + (1.015 * ADAMTS14 exp.) + (0.026 * CSF2 exp.) + (2.973 * ITGA5 exp.), and the risk score for each UVM patient was determined based on the median score using the equation. The optimal number of genes for cross-validation plots was 3, and the selected genes were ADAMTS10, ADAMTS14, and ITGA5.

Further analysis revealed a strong correlation between the expression of the investigated BMRGs and the risk score. The risk score correlation heatmap (Figure 3E) and dot plot (Figure 3F) indicated that the expression levels of ADAMTS10 and ITGA5 were positively correlated with the risk score, while ADAMTS14 was negatively correlated. The TCGA-UVM cohort was used as the training set, and the de-batched GSE84976 dataset was used for validation. In the TCGA-UVM cohort, the low-risk group demonstrated significantly better prognostic outcomes (*p* < 0.001) (Figure 3G). The predictive model showed excellent performance as evidenced by the ROC curves, with high sensitivity and specificity reflected by the AUC values at 1, 2, 3, 4, and 5 years (0.831, 0.916, 0.913, 0.952, and 0.870) (Figure 3H). Moreover, there was an observed increase in mortality and a decrease in survival with higher risk scores (Figure 3I). Principal component analysis (PCA) clearly distinguished low-risk and high-risk patients from each other (Figure 3J). The results obtained in the GSE41613 cohort replicated those of the TCGA-UVM cohort (Figure 3K-N), indicating the reliability and consistency of our predictive model. In conclusion, our prediction model demonstrates high accuracy and reliability, providing valuable guidance for clinical management.

### 3.3 Construction of nomograms based on 3-BMRGs signatures with clinical features

**FIGURE 4:**
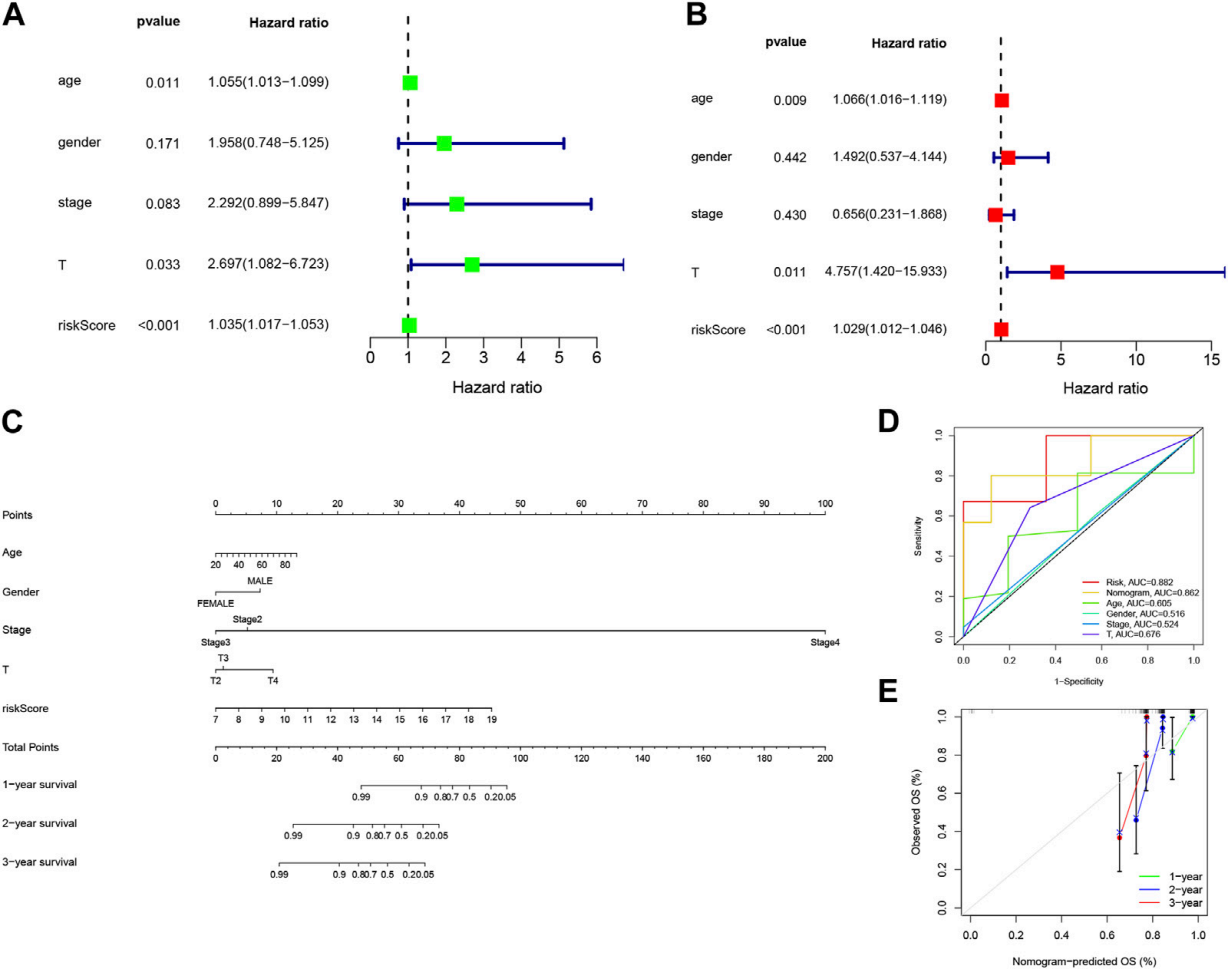
Building nomograms based on clinical characteristics. **(A)** Univariate Cox regression analysis of the signature and various clinical profiles. **(B)** Multivariate Cox regression analysis incorporating the signature and clinical characteristics. **(C)** Nomogram depicting age, gender, stage, T-stage, and risk score. **(D)** Calibration curves of the nomogram for 1-, 3-, and 5-year survival. **(E)** Time-dependent ROC curve.

Given the strong correlation between our constructed risk model and poor prognosis, we conducted univariate and multivariate Cox analyses to determine whether the prognostic characteristics based on the 3-BMRGs could serve as independent predictors of prognosis in UVM patients. In the univariate analysis, age (*p* = 0.011), T-stage (*p* = 0.033), and risk scores (*p* < 0.001) showed significant correlations with prognosis (Figure 4A). The subsequent multivariate analysis confirmed that age (*p* = 0.009), T-stage (*p* = 0.011), and risk scores (*p* < 0.001) remained accurate and independent predictors in this patient cohort (Figure 4B).

To enhance the clinical applicability and usability of the risk model, we developed Nomogram plots that incorporated age, gender, clinical stage, T-stage, and risk scores as predictors of survival probability at 1, 2, and 3 years for UVM patients. The risk score exhibited a substantial impact on predicting overall survival (OS), as demonstrated by the model analysis, indicating that the BMRGs-based risk model could provide more accurate prognostic predictions for UVM patients (Figure 4C). Additionally, we found that the risk score (AUC = 0.882) and Nomogram (AUC = 0.862) outperformed single independent clinical indicators in terms of predictive performance and discriminatory power (Figure 4D). Furthermore, the calibration analysis showed relatively consistent results between the predicted and observed 1-year, 3-year, and 5-year OS rates, as indicated by the calibration line closely aligning with the ideal 45-degree line (Figure 4E).

### 3.4 Clinical correlation and survival analysis of BMRGs in patients with UVM

**FIGURE 5:**
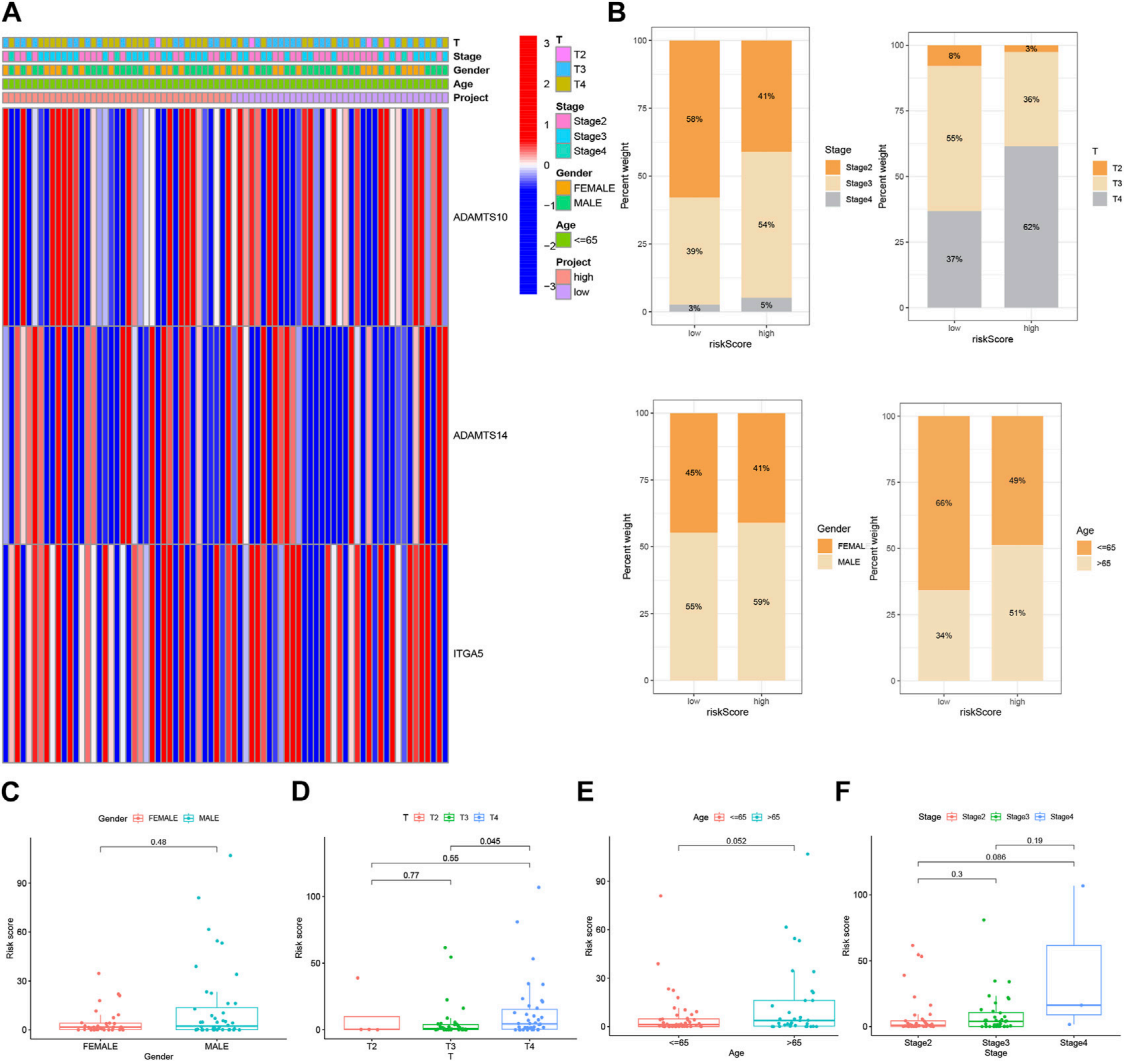
Clinical correlation and survival analysis of BMRGs in UVM patients. **(A)** Heatmap showing the relationship between clinical features and high-risk and low-risk scores in UVM patients. **(B)** Histogram presenting the distribution of clinical characteristics, including stage, T-stage, gender, and age percentages for each category. BMRGs can identify high-risk patients across different subgroups based on various clinicopathological traits. **(C)** Gender, **(D)** T-stage, **(E)** age, and **(F)** stage were analyzed for clinical correlation and survival in UVM patients.

A heat map was generated to visualize the correlation between the prognostic risk model identified using the 3-BMRGs and the clinical characteristics, risk scores, and expression levels of the 3-BMRGs in all UVM patients from the TCGA dataset (Figure 5A). Additionally, we compared the distribution of patients with different clinicopathological features between the high-risk and low-risk groups (Figure 5B). To further examine the association between risk scores and clinicopathological characteristics, box plots were constructed for different subgroups based on gender (male and female), age (>65 and ≤65 years), clinical stage (II and III-IV), and T-stage (T2, T3, T4). Notably, the analysis revealed that patients with stage T4 had significantly higher risk scores compared to those with stage T3 (*p* = 0.045) (Figures 5C–F).

**FIGURE 6:**
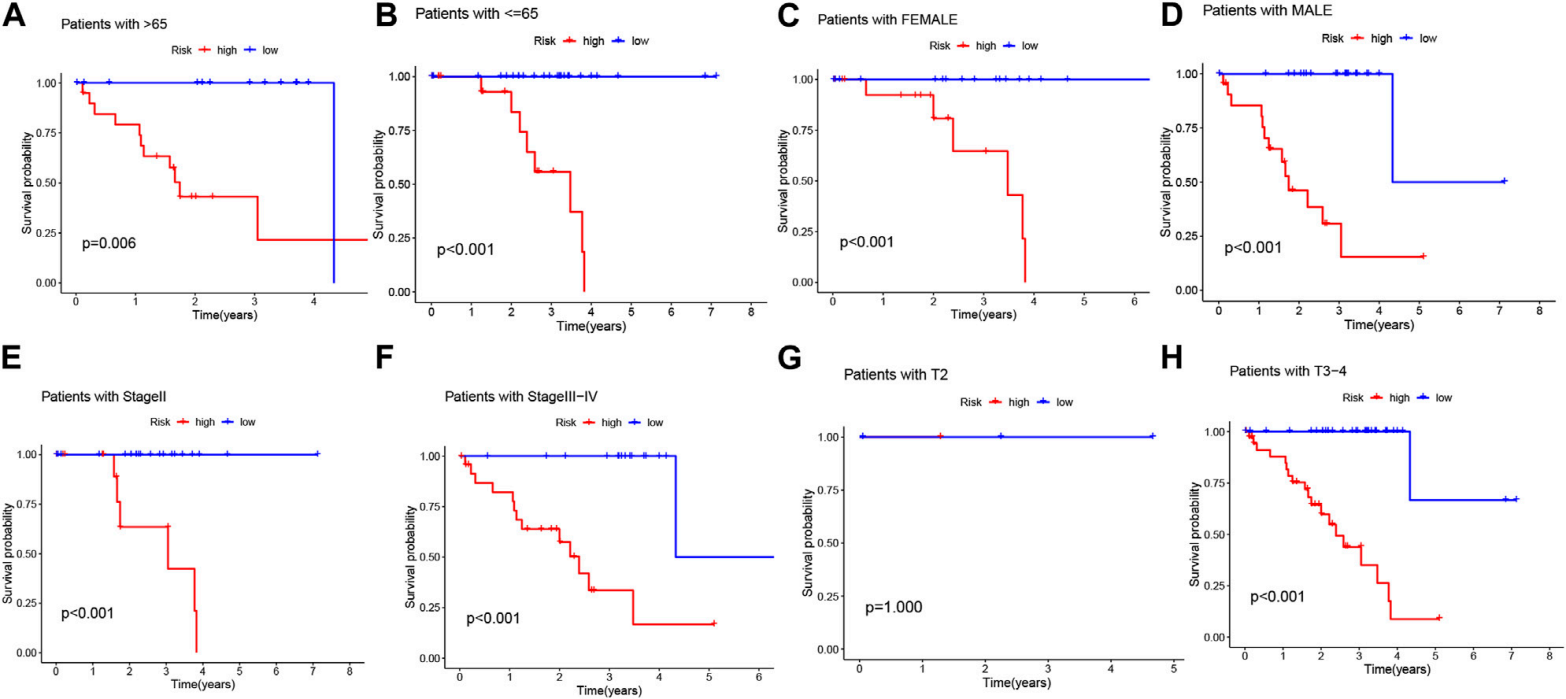
Clinical correlation and survival analysis of 3-BMRGs in UVM patients. **(A, B)** Age, **(C, D)** gender, **(E, F)** tumor grade, and **(G, H)** T-stage evaluated for clinical correlation and survival in UVM patients.

Considering the significant differences in individual clinical characteristics between the high-risk and low-risk groups for overall survival (OS), we further divided UVM patients into subgroups based on age (≤65 years, >65 years), gender (male and female), pathological stage (II and III-IV), and T-stage (T2 and T3-4). Remarkably, except for patients in stage T2, the low-risk subgroup exhibited a significant survival advantage with longer survival times compared to the high-risk subgroup (Figure 6). Based on these analyses, the 3-BMRGs risk model demonstrated its reliability as a clinical prediction tool for UVM patients.

### 3.5 3-BMRGs signatures exhibit superior performance compared to others in prognostic prediction

**FIGURE 7:**
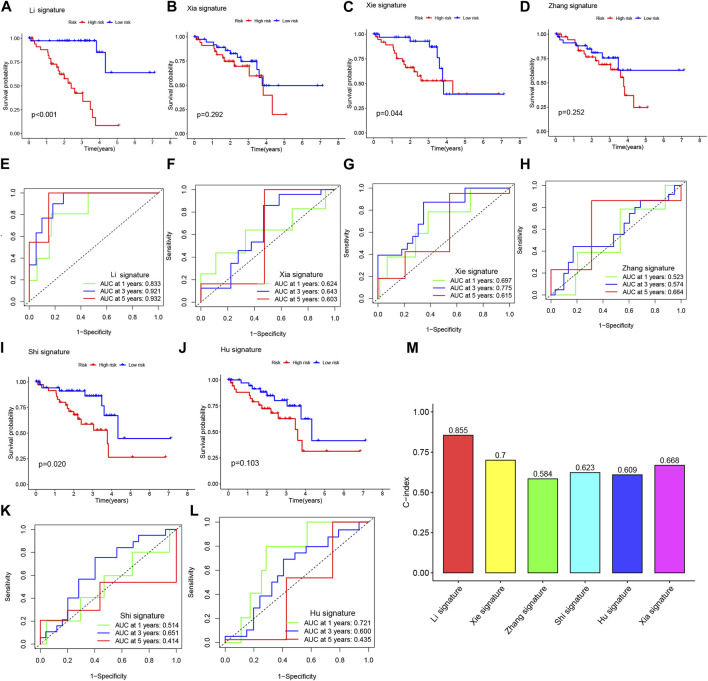
The BMRGs signature demonstrated superior prognostic prediction performance compared to other signatures. **(A, E)** Kaplan-Meier (KM) and receiver operating characteristic (ROC) curves of the BMRGs signature. **(B, F)** KM and ROC curves of the Xia signature. **(C, G)** KM and ROC curves of the Xie signature. **(D, H)** KM and ROC curves of the Zhang signature. **(I, K)** KM and ROC curves of the Shi signature. **(J, L)** KM and ROC curves of the Hu signature. **(M)** C-indexes of the six risk models.

In order to assess the predictive performance of our BMRGs signature in UVM patients, we compared it with five previously published prognostic signatures, namely, the Xia signature, Xie signature, Zhang signature, Shi signature, and Hu signature. Using the same method, we calculated risk scores for each UVM sample in the entire TCGA cohort and found that our signature exhibited the highest correlation with survival outcomes (Figure 7AB, IJ). Despite successfully stratifying UVM patients into two subgroups with significantly different prognoses, the AUC values of the five compared signatures at 1-, 3-, and 5-year survival were lower than those of our model (Figures 7E,F,K,L). Additionally, the C-index analysis demonstrated that our signature outperformed the other signatures (Figure 7M). Overall, our study indicates that our constructed BMRGs signature possesses excellent predictive ability in prognosticating UVM patients.

### 3.6 Functional enrichment analysis of DEGs in TCGA-UVM

**FIGURE 8:**
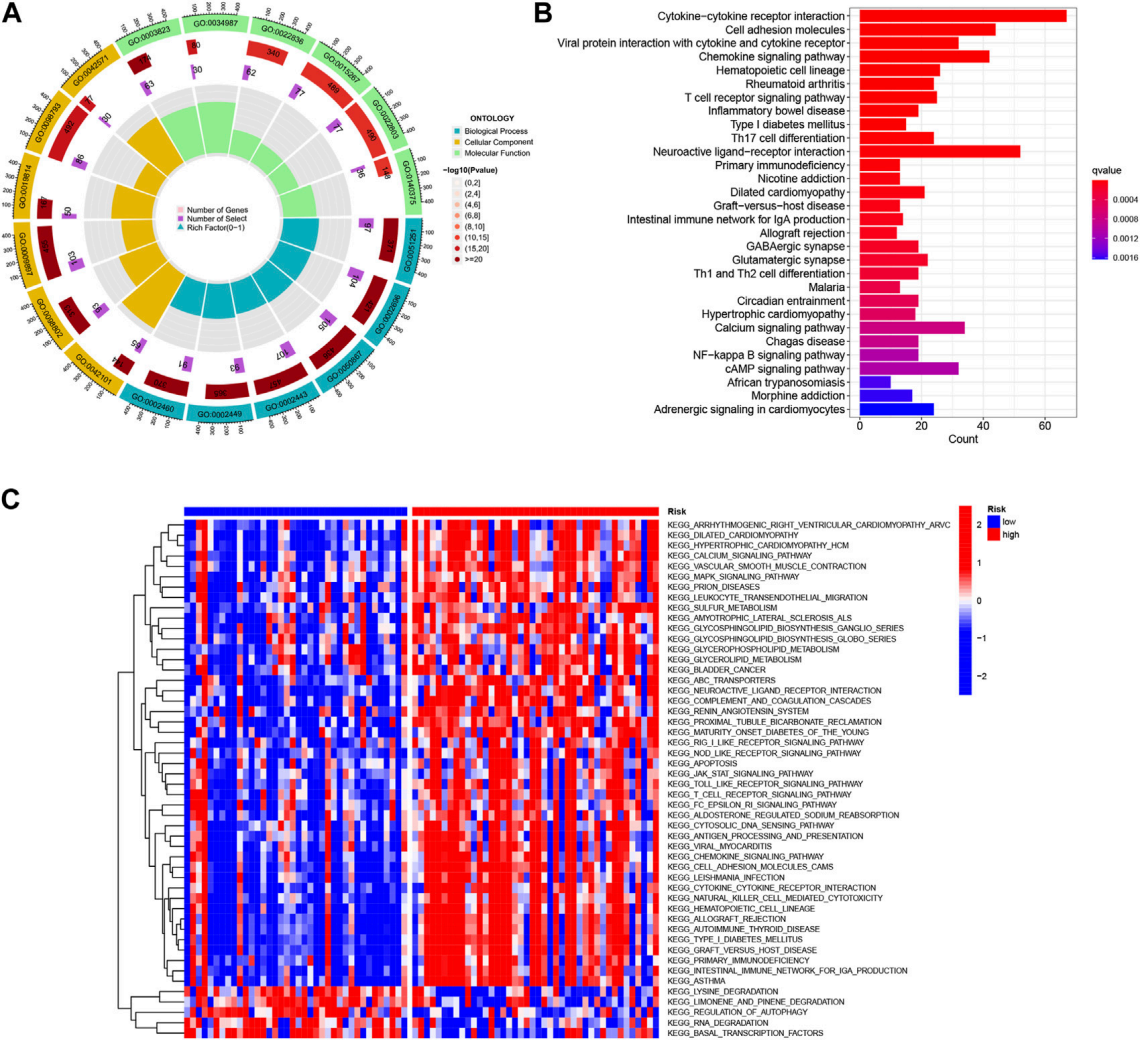
Functional enrichment analysis of Differentially Expressed Genes (DEGs) in TCGA-UVM was performed. **(A)** Gene Ontology (GO) enrichment analysis was conducted to investigate the differences in basement membrane x genes between UVM and normal samples. The analysis included biological processes (BP), cellular components (CC), and molecular functions (MF). **(B)** KEGG enrichment analysis was performed to identify enriched pathways associated with the DEGs. **(C)** Gene Set Variation Analysis (GSVA) was utilized to compare the enrichment scores between high-risk and low-risk cohorts, providing insights into the functional differences between these groups.

In order to gain insights into the potential bioactivities and signaling pathways involved in UVM, and to understand the molecular mechanisms underlying UVM progression, we conducted Kyoto Encyclopedia of Genes and Genomes (KEGG) enrichment analysis and gene ontology (GO) functional analysis. We applied stringent thresholds of FDR<0.05 and *p* < 0.05 to select significantly enriched items, as depicted in Figure 8A and Supplementary Table S1. The biological process (BP) analysis revealed enrichment in various processes such as rhythmic process, regulation of hormone levels, and circadian rhythm. Cellular component (CC) analysis highlighted correlations with neuronal cell body, presynaptic active zone, and terminal bouton, among others. Molecular function (MF) analysis indicated associations with functions such as G protein-coupled receptor binding, receptor-ligand activity, and signaling receptor activator. Furthermore, the KEGG enrichment analysis unveiled disease pathways including Circadian entrainment, Allograft rejection, and the Chemokine signaling pathway (Figure 8B). Moreover, the Gene Set Variation Analysis (GSVA) identified 50 significantly enriched pathways (Figure 8C). In-depth analysis revealed that in the low-risk population, pathways related to the Regulation of autophagy and RNA degradation were enriched. Conversely, in the high-risk population, pathway enrichment primarily involved immune and substance metabolism pathways such as leukocyte transendothelial migration and antigen processing and presentation. These findings contribute to our understanding of the molecular mechanisms underlying UVM and may provide valuable insights for the development of effective therapeutic strategies for UVM patients.

### 3.7 Risk score predicts TME and immune cell infiltration

**FIGURE 9:**
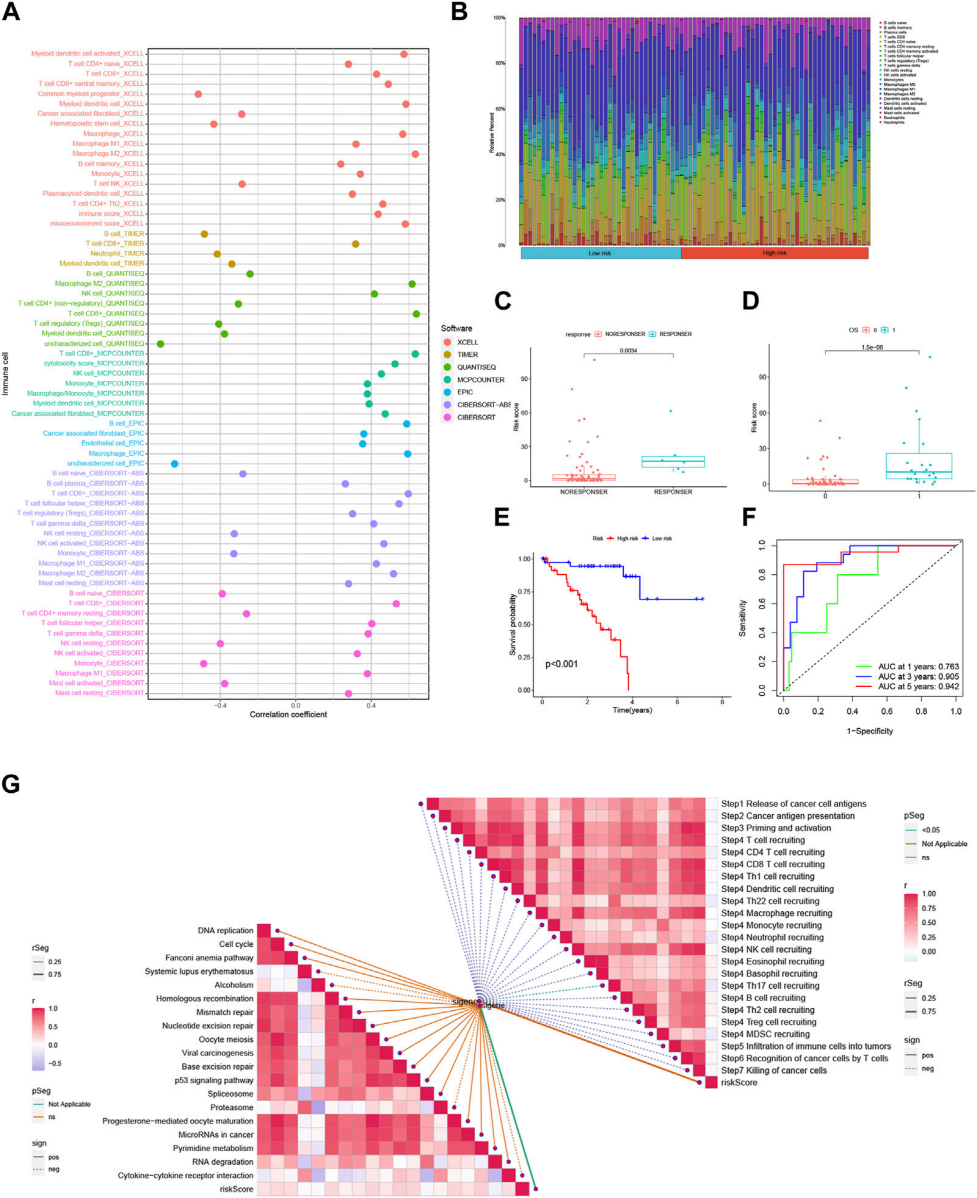
The risk score is predictive of the tumor microenvironment (TME) and immune cell infiltration. **(A)** An immune cell bubble plot was generated to visualize the composition of immune cell types. **(B)** A stacked plot illustrates the differences in immune cell infiltration between the high-risk and low-risk groups. **(C, D)** The expression levels of the 3-BMRGs were utilized to predict the response of patients to immune therapy. **(E)** Kaplan-Meier (KM) curves compare the survival outcomes between the high-risk and low-risk groups after receiving immunotherapy. **(F)** The receiver operating characteristic (ROC) curve analysis demonstrates the robust predictive performance of the marker model. (H) The correlation between risk scores and immune checkpoint blockade (ICB) response characteristics was examined. **(G)** The correlation between risk scores and each step of the tumor immunization cycle was investigated.

Interactions between cancer cells and the tumor microenvironment (TME) play a crucial role in tumorigenesis, progression, and treatment outcomes. Tumor-infiltrating immune cells (TIICs) are integral components of the TME, and their distribution and alterations are closely associated with tumor progression. In this study, we investigated the relationship between risk scores and immune cell infiltration in the context of 3 BMRGs using seven algorithms: XCELL, TIMER, QUANTISEQ, MCPCOUNTER, CIBERSORT, CIBERSORT-ABS, and EPIC. Our results revealed a positive correlation between risk scores and the presence of T cell CD8^+^ cells across multiple algorithms (Figure 9A). Furthermore, we analyzed the proportions of 22 immune cell infiltrates between the high-risk and low-risk groups of TCGA-UVM patients using the CIBERSORT algorithm. The results were visualized using stacked plots, demonstrating differences in immune cell composition between the two risk groups (Figure 9B). We also utilized the immune AI portal to assess immunotherapy response in UVM patients. Our analysis revealed that patients with higher risk scores were more likely to benefit from immunotherapy (Figures 9C,D), while those with lower risk scores exhibited a survival advantage. The ROC curves demonstrated the excellent performance of the 3-BMRGs biomarkers in predicting treatment outcomes for patients (Figure 9F). To further explore the immune profile of the tumor microenvironment, we plotted a correlation butterfly diagram to examine the relationship between risk scores and various steps of the tumor immune cycle. The analysis revealed a positive correlation between risk scores and most immune cycle steps, suggesting potential implications for immune modulation in UVM (Figure 9G).

### 3.8 Relationships between 3-BMRGs signatures and tumor microenvironment

**FIGURE 10:**
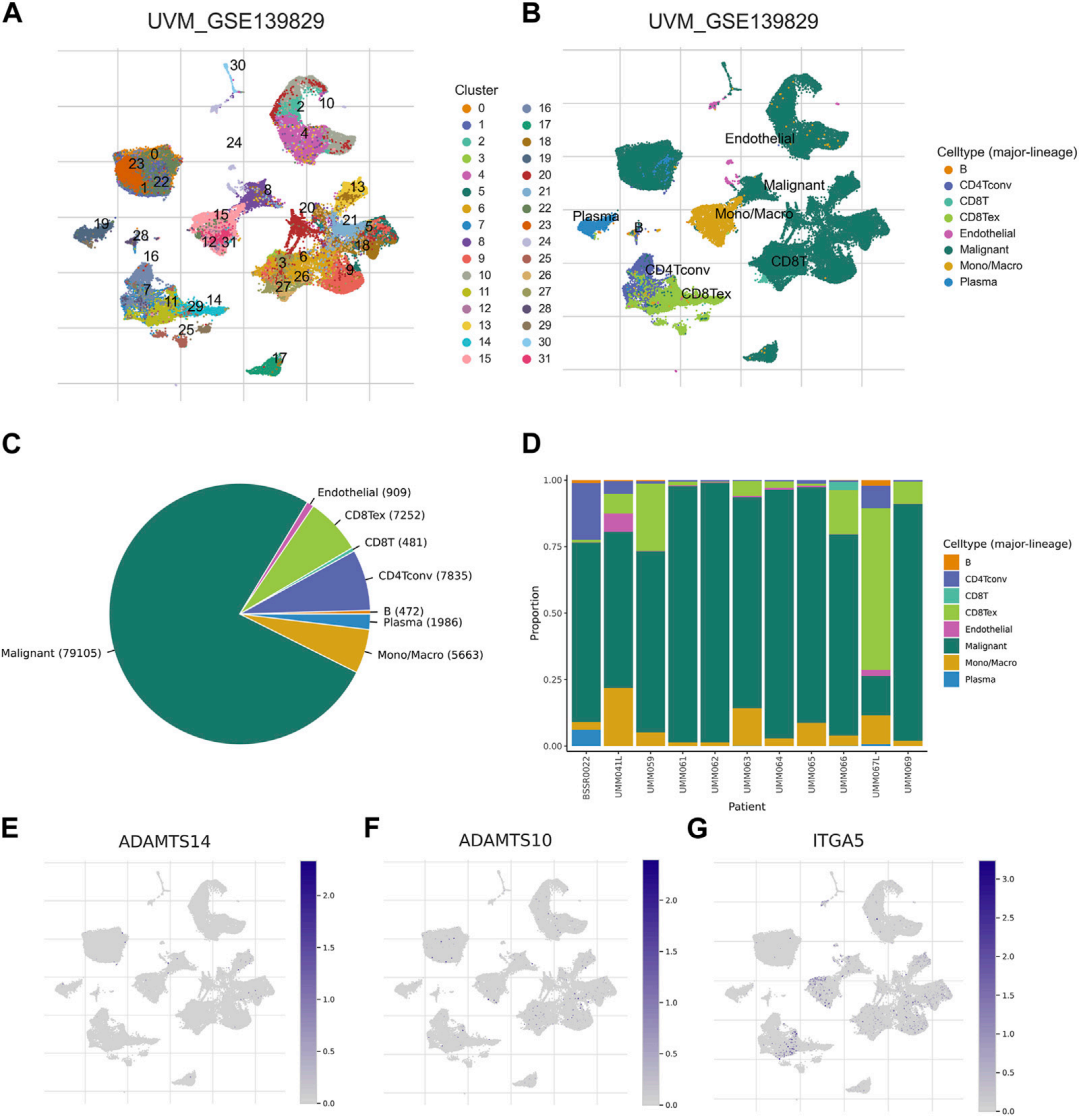
Association of BMRGs with the tumor microenvironment. Annotation of **(A)** 31 cell clusters and **(B)** 8 cell types in UVM_GSE139829. **(C, D)** The distribution and proportion of each cell type, including CD8T cells, endothelial cells, B cells, *etc.* Expression and percentage of **(E)** ADAMTS14, **(F)** ADAMTS10, **(G)** ITGA5.

In order to investigate the expression patterns of the 3-BMRGs in the tumor microenvironment, we utilized the cellular dataset UVM_GSE139829 obtained from the TISCH database. The distribution and numbers of 31 cell populations and 8 immune cell types in the UVM_GSE139829 dataset were analyzed and displayed (Figures 10A–D). Furthermore, we examined the expression of the 3-BMRGs in different immune cell populations. The expression of BMRGs was found to be lower in the ADAMTS14 immune microenvironment (Figure 10E). On the other hand, ADAMTS10 and ITGA5 were expressed in various immune cell populations, as demonstrated in Figures 10F,G, respectively. Notably, ITGA5 showed predominant expression in CD8 Tex, Mono/Macro, and CD8T immune cell populations. These findings provide insights into the expression patterns of the 3-BMRGs within the immune cell landscape of UVM.

### 3.9 ITGA5 facilitates the proliferation, migration, and invasion of uveal melanoma (UVM) cells

**FIGURE 11:**
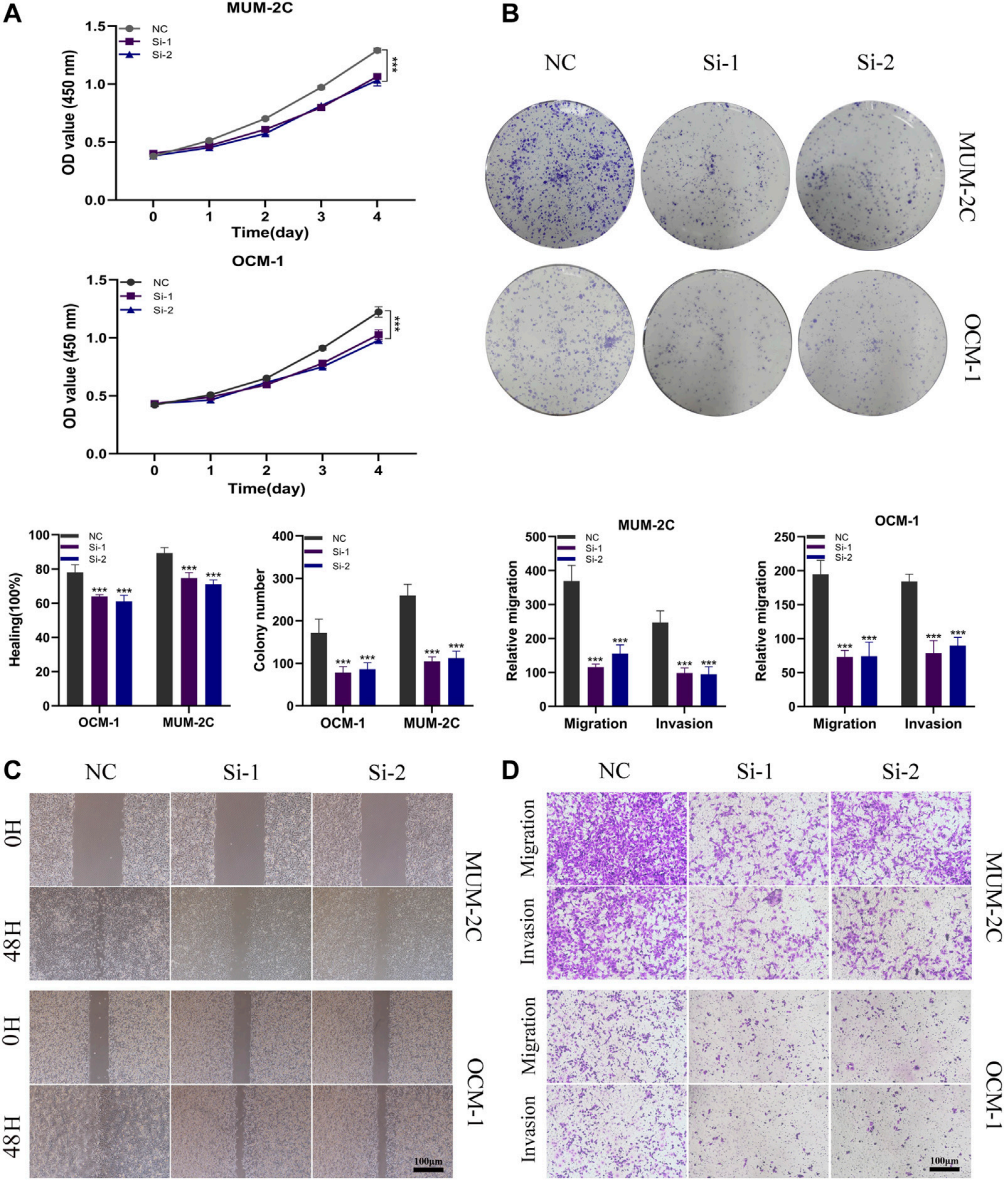
ITGA5 facilitates the proliferation, migration, and invasion of UVM cells. **(A)** CCK-8 assay showed that the proliferative capacity of UVM cells was significantly reduced after silencing of ITGA5. **(B)** Colony formation assays showed that the ability of UVM cells to form colonies was significantly reduced after ITGA5 silencing. **(C, D)** In wound healing and transwell assays, silencing of ITGA5 significantly reduced the migratory and invasive capacity of MuM-2B and OCM-1 cell lines.

Considering the identification of ITGA5 as a high-risk gene exhibiting a maximum absolute hazard ratio (HR) in uveal melanoma (UVM) patients, we conducted additional *in vitro* experiments to elucidate the specific role of ITGA5 in UVM. Knockdown systems targeting ITGA5 were established in OCM-1 and MUM-2C cell lines. The CCK-8 assay, and colony formation assay demonstrated a significant reduction in the proliferation rate of UVM cells following ITGA5 silencing (Figures 11A,B). Moreover, both the Transwell assay and the wound healing assay revealed diminished migration and invasiveness of UVM cells after ITGA5 knockdown, in comparison to cells transfected with si-NC (Figures 11C,D). Collectively, these findings provide evidence that ITGA5 functions as an oncogene, promoting the malignant characteristics of UVM cells, including proliferation, invasion, and migration.

## 4 Discussion

Although the prevalence of UVM is not extremely high, it accounts for 85% of all ocular melanomas, with up to 50% of patients of primary UVM developing distant metastases, 90% with liver damage, and a median survival of 4–5 months (Baggetto et al., 2005; Tabernero, 2007; Carvajal et al., 2017; Brouwer et al., 2019). Additionally, the metastatic rates for UVM throughout the course of 5 and 10 years are roughly 25% and 34%, respectively, and the mortality rate for UVM 1 year after metastasis is 80%. After diagnosis, the majority of patients with metastatic UVM have a survival span of 6–12 months for which metastatic UVM is virtually always challenging to treat. Due to the poor prognosis of UVM, few individuals can receive possibly curative surgery (Singh et al., 2005; Straatsma et al., 2018). Meanwhile, 5-year survival rates have remained essentially unchanged over the past 3 decades despite the development of efficient local therapy. There are currently no effective adjuvant systemic medications that have been proven to lower the risk of metastasis as well as actually extend survival, according to a recent review study (Triozzi and Singh, 2014).

Actually, early diagnosis and therapy are crucial to improving prognosis, while UVM diagnosis and prognosis prediction are currently reliant on clinical presentation and histological examination, which are insufficient to identify tumor heterogeneity and developmental patterns. Therefore, using the TCGA-UVM dataset, this study constructed a multigene prognostic model of genes related to basement membrane proteins from a molecular perspective in order to better predict the diagnosis and prognosis of UVM. It opens up new avenues for the investigation of individualized treatment strategies and prognosis prediction.

Defects in BM protein expression and turnover play a major role in the development of cancer, fibrosis, and diabetes (Tsilibary, 2003; Naba et al., 2014; Foster, 2017). Specifically, the overexpression of laminin, a component of BM protein, is closely associated with the overproliferation of certain tumor cells, such as those found in colon and breast cancer (Jayadev and Sherwood, 2017). Moreover, BM is significantly involved in the progression of tumors. During the early development of breast cancer, cancer cells invade through the BM foramen, which is a crucial step in metastasis (Sikic et al., 2022). In addition, the level of netrin-4 in BM is highly correlated with the prognosis of breast cancer, renal cancer, and uveal melanoma (Reuten et al., 2021). Several recent studies have already attempted to mine public databases to identify prognostic gene signatures related to BM proteins in tumors. For example, Cai et al. identified a 7-gene signature associated with basement membrane proteins that predicted the prognostic status of breast cancer patients and provided insights for immunotherapy (Cai et al., 2022); Zhou et al. developed a risk model using 8 BMRGs, which revealed that clear-cell renal cell carcinoma patients in the low-risk group had a better response to immunotherapy (Zhou et al., 2022); Lin et al. established a 7-BMRG signature and identified five small compounds that could potentially be used for the treatment of pancreatic cancer patients, providing new perspectives for a deeper understanding of this disease (Lin et al., 2023). Overall, these studies highlight the significance of basement membrane proteins in the precise treatment of tumors.

In our study, we selected three basement membrane related-genes (ADAMTS10, ADAMTS14, and ITGA5) to create the novel prognostic model by utilizing Lasso regression analysis, SVM-RFE, and stepwise multiple COX regression analysis. Numerous studies have proven that the ADAMTS (a disintegrin and metalloproteinase with thrombospondin motif) family of proteins contributes to the development of malignant tumors, cell proliferation, apoptosis, migration, invasion, and angiogenesis (Held-Feindt et al., 2006; Rocks et al., 2006; Filou et al., 2015; Sun et al., 2015). ADAMTSs have a negative impact on the prognosis of patients with 24 tumors, in particular the patients with Adrenocortical carcinoma, Uveal Melanoma, Kidney renal clear cell carcinoma, Colon adenocarcinoma, Thyroid carcinoma, *etc.* (Wu et al., 2021). Although the precise mechanism by which ADAMTS play a role in tumor progression and metastasis remains uncertain, several research has explored these protein hydrolases and confirmed their relevance in various tumor types. ADAMTS10 expression is significantly downregulated in tumors (Sun et al., 2015). Furthermore, a variety of ADAMTSs, including ADAMTS20, ADAMTS10, and ADAMTS3, exhibit significant levels of methylation in a range of tumors. Analysis of the relationship between methylation and gene expression levels reveals a negative relationship between the two, suggesting that the main function of ADAMTS methylation is to silence the ADAMTS gene, leading to a decrease in its expression (Wu et al., 2021). Besides, ADAMTS10 is frequently mutated in metastatic colorectal cancer, and mutated ADAMTS10 transcripts are actively expressed in the corresponding tumors implicating a possible role for ADAMTS10 in tumor metastasis (Oga et al., 2019). According to numerous research, the ADAMTS14 gene has been associated with an elevated likelihood of developing tumors. The expression of ADAMTS14 was identified to be considerably higher in human breast cancer tissues, according to Porter et al. (2004). As reported by Sheu et al., ADAMTS14 gene polymorphisms serve a part in the progression of hepatocellular carcinoma (Sheu et al., 2017). The expression of ADAMTS14 in oral squamous carcinoma (OSCC) is low. In OSCC patients, the downregulation of ADAMTS14 may be an effective independent prognostic marker for predicting overall survival because it is predictive of unfavorable clinicopathological characteristics (Lin et al., 2020). Furthermore, there is mounting evidence that Integrin A5 (ITGA5), which plays a major role in the adhesion, migration, and invasion of cancer cells, is highly expressed in several malignancies and contributes to tumor progression (Ohyagi-Hara et al., 2013). One of the markers of invasiveness in head and neck squamous cell carcinoma has been identified as ITGA5 (Yu et al., 2008). Additionally, a study demonstrated that pancreatic ductal malignant adenomas upregulate the ITGA5 gene. Silencing of ITGA5 inhibits the differentiation of human pancreatic stellate cells and reduces connective tissue formation (Kuninty et al., 2019). Furthermore, ITGA5 promotes the development, migration, and invasion of cells that undergo an epithelial-mesenchymal transition in oral cancer (Deng et al., 2019).

The 3-BMRGs we constructed proved to be an independent prognostic factor for UVM. Based on median risk ratings, patients with UVM were separated into high-risk and low-risk groups; there were notable prognostic differences between the two groups. The 3-BMRGs that we created turned out to be a reliable indicator of UVM’s future. Based on median risk ratings, individuals with UVM were separated into high-risk and low-risk groups; there were notable prognostic differences between the two groups. Additionally, evaluations of the ROC and calibration curves revealed that the 3-BMRGs signature had excellent predictive power. To extend the predictive ability of the 3-BMRGs signature and to demonstrate its utility in the prognostic evaluation of UVM patients, we plotted a line graph based on clinical factors and risk scores. Meanwhile, we discovered that the 3-BMRGs signature has better predictive power than clinicopathological features, which could offer clinicians a basis for decision-making.

The tumor microenvironment (TME) is crucial to the metastasis and progression of cancer (Zhao et al., 2022c; Gong et al., 2022; Xiong et al., 2023). The TME comprises cancer cells, surrounding stromal cells, and tumor-infiltrating immune cells, with immune cells playing a dominant role in the TME (Hinshaw and Shevde, 2019; Shen et al., 2022). Through the strengthening of a weakened immune response to tumor cells and the resultant production of an immunological-mediated anti-tumor impact, immunotherapy has made significant strides in the treatment of cancers in recent years (Zhang et al., 2021). As a result, we assessed immune checkpoint expression and discovered that, with the exception of TMIGD2 and CD44, it was highly elevated in the high-risk group of UVM patients. Immune infiltration is closely related to immunotherapy’s efficacy (Nishida and Kudo, 2020). The high-risk group exhibited higher levels of immune cell infiltration, which suggested that they responded more favorably to immunotherapy, according to the ssGSEA enrichment score. Meanwhile, we performed GO and KEGG enrichment analysis to provide more light on the biological pathways and putative molecular mechanisms associated with the BMRG signature. We noted that in the high-risk population, pathway enrichment mainly involved immune and substance metabolism pathways, such as leukocyte transendothelial migration, antigen processing, and presentation. In contrast, pathways related to the regulation of autophagy and RNA degradation were enriched in the low-risk population.

Immune checkpoint blockade has shown significant benefits in the treatment of malignant tumors. Nevertheless, its non-response rate and side effects have posed challenges in clinical practice (Hodi et al., 2010; Kennedy and Salama, 2020; Su et al., 2022). Thus, it is critical to identify individuals who are responsive to different immune checkpoint medicines based on the expression of immune checkpoint genes. Our model has demonstrated excellent results in this regard. In our study, we found that UVM patients with higher risk scores were more likely to benefit from immunotherapy, while patients with lower risk scores could have a higher survival advantage. Furthermore, the TME is closely correlated to the risk model that we built based on the BMRGs.

Our study suffers from the following limitations. Firstly, our study is retrospective, and based on data analysis in public databases with limited inclusion of UVM patients which still demands more clinical data and prospective studies to validate the model and improve the credibility of risk scores; Meanwhile, the extrapolation of our findings is limited due to the possible inherent bias and limitations of the TCGA-UVM and GEO cohorts themselves; In addition, the mechanisms by which BMRGs affect the prognosis of UVM patients are required to be further explored in more *in vivo* experiments.

## 5 Conclusion

To sum up, we have developed a model of the BMRG prognostic signatures including ADAMTS10, ADAMTS14, and ITGA5. Two external validation cohorts were employed to verify the reliability and applicability of the BMRGs scores. This constructed model exhibited robust predictive ability which could act as an independent prognostic factor for UVM, assisting clinicians to identify specific subgroups of patients who may benefit from immunotherapy and chemotherapy, and providing a novel strategy for individualized treatment of UVM patients.

## Data Availability

The datasets presented in this study can be found in online repositories. The names of the repository/repositories and accession number(s) can be found in the article/Supplementary Material.
